# The Effect of Enumeration of Self-Relevant Words on Self-Focused Attention and Repetitive Negative Thoughts

**DOI:** 10.3389/fpsyg.2018.00819

**Published:** 2018-05-29

**Authors:** Seiji Muranaka, Jun Sasaki

**Affiliations:** Department of Clinical Psychology, Graduate School of Human Sciences, Osaka University, Suita, Japan

**Keywords:** self-focused attention, repetitive negative thinking, self-control, limited resources, self-relevant word

## Abstract

Self-focused attention refers to awareness of self-referent, internally generated information. It can be categorized into dysfunctional (i.e., self-rumination) and functional (self-reflection) aspects. According to theory on cognitive resource limitations (e.g., Moreno, 2006), there is a difference in cognitive resource allocation between these two aspects of self-focused attention. We propose a new task, self-relevant word (SRW) enumeration, that can aid in behaviorally identifying individuals’ use of self-rumination and self-reflection. The present study has two purposes: to determine the association between self-focus and SRW enumeration, and to examine the effect of dysfunctional SRW enumeration on repetitive negative thinking. One hundred forty-six undergraduate students participated in this study. They completed a measure of state anxiety twice, before and after imagining a social failure situation. They also completed the SRW enumeration task, Repetitive Thinking Questionnaire, Short Fear of Negative Evaluation Scale, and Rumination-Reflection Questionnaire. A correlational analysis indicated a significant positive correlation between self-reflection and the number of SRWs. Furthermore, individuals high in self-reflection had a tendency to pay more attention to problems than did those high in self-rumination. A significant positive correlation was found between self-rumination and the strength of self-relevance of negative SRWs. Through a path analysis, we found a significant positive effect of the self-relevance of negative SRWs on repetitive negative thinking. Notably, however, the model that excluded self-rumination as an explanatory variable showed a better fit to the data than did the model that included it. In summary, SRW enumeration might enable selective and independent detection of the degree of self-reflection and self-rumination, and therefore should be examined in future research in order to design new behavioral procedures.

## Introduction

Self-focused attention refers to an awareness of self-referent, internally generated information (Ingram, 1990). Self-focused attention has a strong influence over internal information, more so than external information; as such, if the contents of self-focused thoughts are negative, self-focused attention can generate negative affect. For this reason, self-focused attention is considered to play an important role in the development and maintenance of various psychopathological conditions, such as depression, anxiety disorder, and schizophrenia (Spurr and Stopa, 2002). Self-focused attention comprises both functional (self-reflection) and dysfunctional (self-rumination) aspects (Trapnell and Campbell, 1999). Self-rumination refers to self-focused attention motivated by perceived threats, losses, or injustices against the self. When an individual is threatened, they experience anxiety, which in turn causes individuals to pay attention to the enfolding events, their own negative affect, and the consequences of the events. As a result, anxiety can be maintained (e.g., Clark and Wells, 1995). In contrast, self-reflection is self-focused attention motivated by curiosity or an epistemic interest in the self; it reduces depressive mood through encouraging problem-solving behavior during intensely stressful events, which suggests that it facilitates self-regulation to solve problems adaptively (e.g., Mori et al., 2015). Thus, it is clinically important to investigate how to switch from a dysfunctional style to a functional one (e.g., Takano et al., 2012).

However, some studies have shown a significant positive correlation between self-rumination and self-reflection (e.g., Takano et al., 2012; Mori et al., 2015). This finding means that heightening the functional self-focus style ironically results in a similar heightening of the dysfunctional style. Accordingly, methods that independently affect these self-focus styles are needed.

Individuals’ motivation for focusing on the self and limitations in their cognitive resources potentially determine whether they adopt a functional or dysfunctional self-focus style. Sweller (1988) proposed cognitive load theory (CLT) to explain how limited cognitive resources influence selective attention. According to CLT, depletion of limited cognitive resources decreases performance on certain tasks (in terms of achievements such as number of errors and time spent on the task) (Paas et al., 2003). High ruminator depletes attentional resources for problem solving, and has difficulty in discarding no-longer-relevant negative words from working memory (Koster et al., 2011; Zetsche et al., 2012). The degree to which one allocates these limited cognitive resources to the self in turn may define the style of self-focused attention (Whitmer and Gotlib, 2013). Labor motivation, which orient “have to” goal lead to depleted limited cognitive resources. It need to switch to leisure motivation, which orient “want to” goal (Inzlicht et al., 2014).

Self-regulation and motivation appear to mediate the selection, organization, and integration of information in working memory, as well as retrieval from long-term memory. Motivation also appears to mediate selective attention of information (Moreno, 2006), which may lead to a difference in the allocation of resources between the functional and dysfunctional self-focus style. A ruminative self-focus makes individuals narrowly focus on negative information when negative affects occur (Whitmer and Gotlib, 2013), whereas a self-reflective focus is associated with distancing oneself from problems and paying greater attention to the more positive aspects of the self because self-reflection tends to lead to calmer responses to negative experiences (Ayduk and Kross, 2010). Self-regulation is also a relevant factor; it refers to the capacity to override one’s thoughts, feelings, and habitual patterns of behavior. High self-regulation can control negative thoughts such as death related thoughts (Galliot et al., 2006). Baumeister and Vohs (2007) noted that motivation can be helpful in overcoming ego depletion (i.e., a state of depleted cognitive resources), although it must be restrained in order to maintain self-regulation. Thus, motivation is an important influencing factor of self-regulation and how people control intensive negative thoughts. Furthermore, it is crucial in learning to utilize limited cognitive resources for functional self-focused attention.

According to Muraven et al. (1998), cognitive resources can be measured using the time spent engaging in a behavioral task—particularly, how long an individual attends to self-relevant information in such a task. We strove to develop a behavioral task in order to assess self-focused attention through the enumeration of words that individuals use to describe themselves. This task, called self-relevant word (SRW) enumeration, was designed to enable identification of a current process of self- rumination and self-reflection. As mentioned above, negative emotions narrow the attentional scope and maintain rumination (Whitmer and Gotlib, 2013). Furthermore, Gaebler et al. (2014) found a correlation between self-relatedness and the intensity of negative emotion among patients with social anxiety disorder who were looking at aversive pictures, and they reacted more strongly to negative information when the intensity of their negative emotions was high. Accordingly, we expected that individuals with higher self-rumination might better retain negative words. In other words, the strength of the relationship with negative SRWs might be usable as an index of self-rumination. We predicted that self-rumination is associated with the strength of the relevance of negative SRWs (negative SRWstr). On the other hand, individuals higher in self-reflection were expected to have more comprehensive self-perceptions, which means that they would produce a higher number of SRWs overall (SRWnum). This is based on the findings of Ayduk and Kross (2010), who noted that individuals higher in self-reflection tend to adopt a broader perspective on themselves.

Numerous studies have begun focusing on the function of repetitive negative thinking (RNT) in order to understand emotional upsets and persistent emotional disorders (Kashdan and Roberts, 2007; Ehring and Watkins, 2008). RNT refer to iterative thinking about negative content. We assume that SRW enumeration is related to RNT. Confirming the effect of a dysfunctional SRW enumeration style on RNT can help in developing techniques to decrease RNT in emotional disorders.

The present study had two purposes: to determine the association between self-focus and SRW enumeration, and to confirm the effect of dysfunctional SRW enumeration on RNT. We formulated and tested three hypotheses in relation to these purposes. First, there is a positive correlation between self-reflection and the SRWnum. Second, there is a positive correlation between self-rumination and the negative SRWstr. Finally, based on the idea that rumination is maintained by narrowing the attentional scope to the self (Whitmer and Gotlib, 2013), negative SRWstr is expected to have a direct effect on RNT.

## Materials and Methods

### Participants

A total of 146 undergraduate students participated (49 males and 97 females) in this study. Their mean age was 20.92 years [standard deviation (SD) = 3.30].

### Measures

#### State-Trait Anxiety Inventory (Japanese Version; Shimizu and Imae, 1981)

Participants completed the Japanese version of the state scale of the State-Trait Anxiety Inventory (STAI-S) before and after imaging a situation (see below). The STAI-S measures the degree of state anxiety; it contains 20 items and participants are required to answer each item on a 4-point Likert-type scale from 1 (not at all) to 4 (very much). A Cronbach’s alpha of 0.87 was reported, indicating high validity (Shimizu and Imae, 1981). We termed scores on the STAI-S before and after the imagining a social failure situation as pre-STAI-S and post-STAI-S scores, respectively. We also calculated the extent to which anxiety increased, denoted as the increment in STAI-S score.

#### Short Fear of Negative Evaluation (Japanese Version; Sasagawa et al., 2004)

Participants completed the short fear of negative evaluation (SFNE), which measures the degree to which individuals fear negative evaluations. This questionnaire contains 12 items, each rated on a 5-point Likert-type scale ranging from 1 (strongly disagree) to 5 (strongly agree). Sasagawa et al. (2004) confirmed that this scale has high measurement precision according to item response theory. In this study, we increased participants’ level of state anxiety by having them imagine a social failure situation. Thus, the SFNE was used to confirm whether fear of negative evaluation (also called social trait anxiety) affected the increase in state anxiety after the imagination task.

#### Repetitive Thinking Questionnaire (Japanese Edition; Tanaka and Sugiura, 2014)

Participants completed the Japanese version of the Repetitive Thinking Questionnaire (RTQ), which measures their degree of RNT. This questionnaire contains 10 items, each rated on a 5-point Likert-type scale ranging from 1 (strongly disagree) to 5 (strongly agree). The scale had a Cronbach’ s alpha value of 0.91, indicating high reliability (Tanaka and Sugiura, 2014).

#### Rumination-Reflection Questionnaire (Japanese Edition; Takano and Tanno, 2008)

We used the Rumination-Reflection Questionnaire (RRQ) to assess participants’ self-rumination and self-reflection tendencies. This questionnaire contains 24 items (two subscales with 12 items each), each rated on a 5-point Likert-type scale ranging from 1 (strongly disagree) to 5 (strongly agree). Takano and Tanno (2008) found that the scale had good internal consistency (α = 0.89 for self-rumination; α = 0.89 for self-reflection).

### Situation Imagination Task

Participants were asked to imagine a social failure situation after reading text in Japanese taken from a study by Fujii (2013): “You have broken some dishes in front of a large number of guests in a restaurant.” Participants were asked to report the total number of guests and the number of guests who noticed the sound of the breaking dishes.

### SRW Enumeration Task

In this task, participants were asked to enumerate and describe as many SRWs as they could, as outlined in the following instructions (in Japanese): “Please write as many words that you can use to describe yourself as you can.” After enumerating the words, participants had to indicate the emotional valence and self-relevance of each word. Emotional valence was measured using a 5-point Likert-type scale, ranging from 1 (negative) to 5 (positive). Therefore, SRWs with an emotional valence scored 1 or 2 were regarded as negative. Self-relevance was also evaluated using a 5-point Likert-type scale, ranging from 1 (strongly disagree) to 5 (strongly agree). In this study, participants could write down a maximum of 16 SRWs because of the limited available space on the page. Examples of SRWs written by participants include “ashamed,” “anxious,” “impatient,” and so on. We calculated two scores from the SRWs: the number of SRWs (SRWnum) and the strength of their self-relevance (SRWstr).

### Procedure

All participants were recruited from introductory psychology classes. With the lecturer’s cooperation, a researcher distributed the questionnaires and written instructions about this study to participants. Participants completed the STAI-S twice, before and after the situation imagination task. Next, they completed the SRW enumeration task and the RTQ, SFNE, and RRQ. Participants spent approximately 10 min in total. No incentive was given to any participants.

The study protocol was approved by the institutional review board of an author’s affiliated institution and this study was carried out in accordance with the recommendations of the ethics committee of the Graduate School of Human Sciences, Osaka University. Written informed consent was obtained from all subjects in accordance with the Declaration of Helsinki.

### Statistical Analysis

The normality of each variable was tested before any further analysis. A skewness of over 2.0 and a kurtosis of over 7.0 were considered to reflect a moderately non-normal distribution (Curran et al., 1996). Our analysis consisted of three parts. First, the discrepancies between pre- and post-STAI-S scores were examined with a paired *t*-test. Second, hypotheses 1 and 2 were investigated via correlational analyses between self-reflection scores and SRWnum, as well as between self-rumination scores and negative SRWstr. The correlational analysis required a sample size of at least 80 for a middle effect size and sufficiently high power [ρ = 0.3, degree of power (1-β) = 0.80]. Therefore, this study had a sufficiently large sample. Finally, hypothesis 3 was examined via a path analysis to confirm the relationship between SRW enumeration and RNT. The effect of self-rumination was nested if hypothesis 1 was followed. Moreover, the effect of the degree of increase in anxiety was nested because negative emotional intensity has a positive effect on self-relatedness (Gaebler et al., 2014).

## Results

### Descriptive Statistics and Manipulation Check

Descriptive statistics are shown in **Table 1**. The SRWnum ranged from 0 to 16, while the negative SRWnum ranged from 0 to 11. The overall and negative SRWstr ranged from 0 to 5. A normality test revealed that the kurtosis of SRWnum was over 7.0. Thus, it was regarded as having a non-normal distribution.

**Table 1:** Descriptive statistics and internal consistency.

	*Mean*	*SD*	*Chronbach’s α*	*Skewness*	*Kurtosis*
Pre-STAI-S	2.12	0.29	0.92	0.60	2.92
Post-STAI-S	2.35	0.39	0.94	0.47	2.59
Increment in STAI-S	0.23	0.53	–	1.21	6.16
SRWnum	4.08	8.07	–	2.17	9.75
Negative SRWnum	2.86	2.00	–	1.46	5.79
SRWstr	4.07	0.77	–	-1.06	4.14
Negative SRWstr	4.09	0.85	–	-1.07	3.86
Repetitive Thinking Questionnaire	2.84	0.82	0.90	-0.04	2.24
Shot fear of negative evaluation scale	3.61	0.80	0.93	-0.53	2.24
Self-rumination	3.61	0.64	0.91	-0.45	2.61
Self-reflection	3.37	0.57	0.88	0.11	2.34

STAI-S, State-Trait Anxiety Inventory – State; pre, measurement before situation imagination; post, measurement after situation imagination; SRW, self-relevance word.

As a manipulation check, a paired *t*-test was used to examine the discrepancy between pre-STAI-S and post-STAI-S scores. We found that the post-STAI-S score was significantly higher than was the pre-STAI score [*t*(284.35) = 3.32, *p* < 0.001; *ES*: *Cohen’s d* = 0.70, 1-β > 0.99]. The results of the correlational analysis (shown below) indicated significant correlations between scores for the SFNE and each administration of the STAI-S. This finding indicates that fear of negative evaluation could be influenced by the increment in anxiety. Thus, a correlation was calculated between the increment in STAI-S scores and the SFNE scores. This was not significant (*r* = 0.09, n.s.).

### Correlation Matrix

**Table 2** shows the correlation matrix. Significant positive correlations were found between the pre-STAI-S scores and the post-STAI-S (*r* = 0.58, *p* < 0.001), RTQ (*r* = 0.38, *p* < 0.001), SFNE (*r* = 0.35, *p* < 0.001), and self-rumination scores (*r* = 0.39, *p* < 0.001). Post-STAI-S scores were also significantly and positively correlated with all other variables (*rs* = 0.17–0.46, *ps* < 0.05).

**Table 2:** Correlation matrix.

	1	2	3	4	5	6	7	8	9	10
1. Pre-STAI-S	–									
2. Post-STAI-S	0.58***	–								
3. Increment in STAI-S	-0.33***	0.57***	–							
4. SRWstr	0.03	0.19*	0.20	–						
5. Negative SRWnum	0.09	0.20*	0.14	-0.10	–					
6. Negative SRWstr	0.09	0.27*	0.22**	0.78***	0.06	–				
7. Repetitive Thinking Questionnaire	0.38***	0.46***	0.16	0.13	0.05	0.21*	–			
8. Short fear of negative evaluation	0.35***	0.38***	0.09	0.04	0.12	0.16	0.39***	–		
9. Self-rumination	0.39***	0.46***	0.13	0.12	0.07	0.21*	0.48***	0.66***	–	
10. Self-reflection	0.03	0.17*	0.17	-0.06	0.13	0.04	0.15	0.21*	0.39***	–
11. SRWnum	0.12	0.06	-0.02	-0.16*	0.71***	-0.01	0.04	0.02	0.02	0.20*

^∗^*p* < 0.05, ^∗∗^*p* < 0.01, ^∗∗∗^*p* < 0.001; STAI-S, State - Trait Anxiety Inventory – State; pre, measurement before situation imagination; post, measurement after situation imagination; SRW, self-relevant word. The correlation between each variable and SRWnum calculated with Spearman’s method (ρ).

The increment in STAI-S score was significantly and positively correlated with SRWstr (*r* = 0.20, *p* < 0.05) and negative SRWstr (*r* = 0.22, *p* < 0.01). There was also a significant positive correlation between SRWstr and negative SRWstr (*r* = 0.78, *p* < 0.01). Negative SRWstr was significantly and positively correlated with RTQ (*r* = 0.21, *p* < 0.05) and self-rumination scores (*r* = 0.21, *p* < 0.05). Positive correlations were observed between RTQ scores and SFNE (*r* = 0.39, *p* < 0.001) and self-rumination scores (*r* = 0.48, *p* < 0.001). SFNE scores were positively correlated with self-rumination (*r* = 0.66, *p* < 0.001) and self-reflection scores (*r* = 0.21, *p* < 0.05). A significant positive correlation was also found between self-rumination and self-reflection (*r* = 0.39, *p* < 0.001).

As the SRWnum had a non-normal distribution, we evaluated its correlations with each variable via Spearman’s coefficient. SRWnum had positive correlations with negative SRWnum (ρ = 0.71, *p* < 0.001) and self-reflection scores (ρ = 0.20, p < 0.05), and negative correlations with SRWstr (ρ = -0.16, *p* < 0.05). Thus, hypothesis 1 (i.e., self-reflection has a positive correlation with SRWnum) was partially supported by our results. There was no correlation between self-rumination and negative SRWnum. This finding, coupled with the above results concerning the positive relation between negative SRWstr and self-rumination, was supportive of hypothesis 2 (i.e., there is a positive correlation between self-rumination and the strength of self-relevance of negative SRWs). Moreover, the correlation between self-rumination and self-reflection (*r* = 0.39) was significantly stronger than was the correlation between SRWnum and negative SRWstr (ρ = -0.01; *z* = 3.39, *p* < 0.001). Therefore, negative SRWstr might reflect the degree of self-rumination.

### The Effect of SRW Enumeration on RNT (**Figure 1**)

**FIGURE 1:**
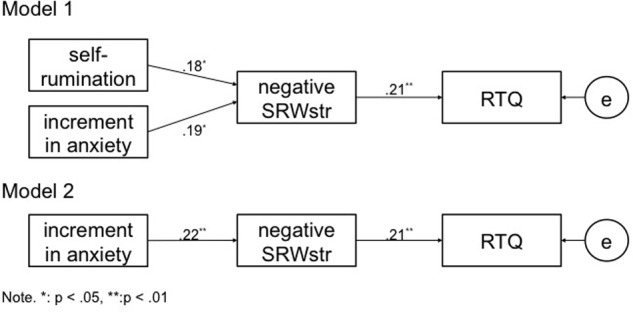
Two models of the effects on RTQ.

A path analysis was used to examine the statistical effect of negative SRWstr on RNT. The above correlational results indicate positive correlations of negative SRWstr with self-rumination and the increment in anxiety. Thus, Model 1 focused on the path from self-rumination to increment in STAI-S scores, negative SRWstr, and RTQ scores. Model 1 did not show a good fit to the data, as the goodness-of-fit indices did not meet the required standards: χ^2^(3) = 37.50, *p* < 0.001; goodness of fit index [GFI] = 0.89; adjusted GFI [AGFI] = 0.64, root mean square error of approximation [RMSEA] = 0.28, Akaike information criterion [AIC] = 51.50. Because the effect of self-rumination on negative SRWstr was somewhat weaker than was the effect of the increment in anxiety, self-rumination was removed from Model 1 (thereby creating Model 2). The results for Model 2 indicated a good fit to the data based on the following fit indices: χ^2^(1) = 1.89, n.s.; GFI = 0.99, AGFI = 0.95, RMSEA = 0.08, and AIC = 11.89.

## Discussion

This study aimed to determine (1) the association between self-focus and SRW enumeration, and (2) the effect of dysfunctional SRW enumeration on RNT. The hypotheses were as follows: (1) self-reflection is positively correlated with SRWnum; (2) self-rumination is positively correlated with negative SRWstr; and (3) negative SRWstr has a significant effect on RNT.

The increase in STAI-S scores revealed that our anxiety manipulation was effective. Moreover, there was no significant correlation between increment in STAI-S scores and SFNE scores, indicating that the situation imagination task led to an increase in anxiety independent of participants’ social trait anxiety.

The correlational analysis revealed a significant positive correlation between self-reflection and SRWnum. However, we cannot conclude that this result fully supports hypothesis 1 because SRWnum had a non-normal distribution. Individuals with high self-reflection have a tendency to pay greater attention to their own problems than do those with high self-rumination (Ayduk and Kross, 2010), which potentially explains their greater SRW enumeration. Moreover, there was a significant negative correlation between SRWnum and SRWstr. This finding suggests that paying greater attention to the self might lower the self-relevance of each piece of information. However, this association between SRW enumeration and attention to the self cannot be explained by the current results, and thus should be investigated further. As noted in section 1, motivation is a mediating factor underlying the integration of self-information in working memory and retrieving self-information from long-term memory (Moreno, 2006). Therefore, the number of SRW refer to easiness accessing to self-information in long-term memory. It is assumed that the association between SRW enumeration and processing of self-information can be investigated in future research, and that understanding this connection would clarify why self-reflection is functional.

On the other hand, we observed a significant positive correlation between self-rumination and negative SRWstr, which supports hypothesis 2. Trait ruminators have difficulty in discarding no-longer-relevant negative words from working memory (Zetsche et al., 2012). This tendency is considered to reflect a difficulty in switching attention away from negative information, and is the reason for the narrowed attentional scope among ruminators (Whitmer and Gotlib, 2013). Koster et al. (2011) pointed out that trait ruminators have fewer attentional resources for information that is not negative. Based on our findings, we can assume that trait ruminators allocate their limited attentional resources primarily to negative information about themselves, which increased their negative SRWstr. Negative SRWstr was also positively correlated with the increment in state anxiety. A previous study indicated that the intensity of negative emotion influences self-relatedness (Gaebler et al., 2014). Although this study used SRWs enumerated by the participants, whereas Gaebler et al. (2014) measured the intensity of negative emotion and self-relatedness using pictures, increasing negative emotion induce linking with negative information. Thus, we can conclude that increased negative emotion to some degree correlates with the strength of the self-relevance of information.

Additionally, we examined whether the correlation between self-rumination and self-reflection was stronger than was the correlation between SRWnum and negative SRWstr to confirm the reliability of the SRW enumeration task. Some reports have shown a significant positive correlation between self-rumination and self-reflection (e.g., Takano et al., 2012; Mori et al., 2015). Our results indicate individuality between SRWnum and negative SRWstr. Therefore, investigating self-focus using SRW enumeration may allow us to find ways of selectively increasing only self-reflection.

In addition to Trapnell and Campbell’s (1999) approach to classifying self-focus (which is based on motivation for self-focus, i.e., self-rumination vs. self-reflection), Teasdale (1999) has attempted to classify self-focus by style of focus: experiential self-focus and analytical thinking. Experiential self-focus is functional, and is induced by focusing on the experience of internal feelings. Analytical thinking refers to a dysfunctional form of self-focus that is produced by excessive thinking about the causes, consequences, and the meaning of internal feelings. Based on this theory, we assume that the tendency in experiential self-focusing to concentrate on self-information exploration without evaluation is associated with the positive correlation found between self-reflection and SRWnum, and with the lack of correlation between SRWnum and SRWstr in this study. On the other hand, the tendency in analytical thinking to focus on self-information while making evaluations perhaps underlies the positive correlation between self-rumination and negative SRWstr.

The results of our study indicate that SRW enumeration might support both of the theories of self-focus—Teasdale’s (1999) or Trapnell and Campbell’s (1999)—and help integrate them. To some extent, however, the practical implications of our results might differ according to the theory. If Teasdale’s (1999) theory is supported, interventions by clinicians might need to address self-focus according to experiential style. On the other hand, if Trapnell and Campbell’s (1999) theory is supported, interventions for self-focus need to focus on curiosity and motivation.

The results of the path analysis indicate a significant positive effect of negative SRWstr on RNT, which supports hypothesis 3. However, the model that excludes self-rumination as an explanatory variable demonstrated a better fit to the data than did the model that included it. This result may reflect how, because self-rumination is a trait, it does not respond to changes in state measurements. Retrospective assessments, such as those measuring traits, tend to show negative bias (Moberly and Watkins, 2008). SRW enumeration is regarded as a real-time state measurement; hence, it might respond to self-related statements more than trait measurements. Therefore, it is noted that the association between self-rumination and effect of negative SRWstr on RNT is needed to examine with the process of self-rumination measurement. Our result reveals that increasing anxiety has a positive effect on negative SRWstr, which is in turn connected with the positive effect on RNT. It is expected that a method of investigating negative SRWstr would decrease negative thinking when anxiety occurs.

In summary, SRW enumeration might enable selective detection of the degree of self-reflection and self-rumination. Moreover, the model indicated that increasing anxiety influenced negative SRWstr and RNT. This model suggests that it is enable to decrease RNT to investigate whether negative SRWstr is lower when anxiety is higher. In contrast, to improve self-reflection, it might be necessary to devise ways of enhancing SRWnum; this could lead to more adaptive problem-solving behavior and decreased depression. (Mori et al., 2015). These associations should be examined in future research, as they might enable new procedures using SRW enumeration.

This study has four main limitations. First, we did not include a group that did not experience the anxiety manipulation. This was because the purpose of this study was to determine how SRW enumeration helps in identifying different categories of self-focus. However, because the effect of the increment in anxiety was associated with SRW measurements, future studies should incorporate a control group with no anxiety manipulation. Second, although the manipulation of anxiety by situation imagination was valid, future research should use a method more closely resembling daily life. Third, we measured SRWs using a questionnaire because we could not control the time spent on SRW enumeration, and there were some individual differences in response time. This suggests there were some discrepancies in meaning among participants, even when they had the same number of SRWs; this difference might have affected the results. Thus, response time should be controlled experimentally in future studies. Finally, we could not identify any factors that were negatively correlated with SRW enumeration and self-focused attention. Examining the factors related to decreased ruminative self-focus or RNT is important for future studies, particularly in terms of the treatment of mental disease such as depression and anxiety disorder.

## Author Contributions

SM contributed to the conception and design of the study, analysis and interpretation of the results, and writing the manuscript. JS contributed to the conception of the study and revising the manuscript.

## Conflict of Interest Statement

The authors declare that the research was conducted in the absence of any commercial or financial relationships that could be construed as a potential conflict of interest.
